# Stereotactic Ablative Radiotherapy for Oligometastatic Disease in Liver

**DOI:** 10.1155/2014/340478

**Published:** 2014-04-29

**Authors:** Myungsoo Kim, Seok Hyun Son, Yong Kyun Won, Chul Seung Kay

**Affiliations:** Department of Radiation Oncology, Incheon St Mary's Hospital, College of Medicine, The Catholic University of Korea, 56 Dongsu-ro, Bupyeong-gu, Incheon-si, Seoul 403-720, Republic of Korea

## Abstract

Liver metastasis in solid tumors, including colorectal cancer, is the most frequent and lethal complication. The development of systemic therapy has led to prolonged survival. However, in selected patients with a finite number of discrete lesions in liver, defined as oligometastatic state, additional local therapies such as surgical resection, radiofrequency ablation, cryotherapy, and radiotherapy can lead to permanent local disease control and improve survival. Among these, an advance in radiation therapy made it possible to deliver high dose radiation to the tumor more accurately, without impairing the liver function. In recent years, the introduction of stereotactic ablative radiotherapy (SABR) has offered even more intensive tumor dose escalation in a few fractions with reduced dose to the adjacent normal liver. Many studies have shown that SABR for oligometastases is effective and safe, with local control rates widely ranging from 50% to 100% at one or two years. And actuarial survival at one and two years has been reported ranging from 72% to 94% and from 30% to 62%, respectively, without severe toxicities. In this paper, we described the definition and technical aspects of SABR, clinical outcomes including efficacy and toxicity, and related parameters after SABR in liver oligometastases from colorectal cancer.

## 1. Introduction

Colorectal cancer (CRC) is the third most commonly diagnosed malignancy and the second leading cause of cancer-related death in the USA [1]. Approximately 15%–25% of CRC patients have liver metastases at the time of diagnosis, and liver metastases develop within 3 years in another 25%–50% of patients with CRC following resection of the primary tumor [2]. The median survival time of these patients ranges from 5 to 12 months without treatment [3]. For patients with metastatic CRC, systemic therapy, including chemotherapy and molecular-targeted agents, is preferred and the development in systemic therapy has led to prolong survival [4]. However, systemic therapy itself rarely eradicates sites of macrometastatic disease permanently. For some subset of patients with a finite number of discrete lesions in the liver, surgical resection has led to 5-year survival rates of up to 60% in some recent series [5–7]. In this “oligometastatic” setting, local therapy has the potential to cure liver metastases, improving survival. Unfortunately, 80%–90% of patients with liver metastases are not suitable for surgical resection due to unfavorable tumor factors or medical comorbidities. Nonsurgical approaches, such as radiofrequency ablation (RFA), transarterial chemoembolization, and cryotherapy, have been widely used as alternative local therapy for unresectable liver metastases. However, each of these techniques has limitations such as the size, the location, the number of tumors, and the variable high recurrence rates [8–10].

Historically, the radiation therapy has had a limited role in the treatment of liver cancer due to the low tolerance of the liver to radiation dose and it was difficult to deliver the radiation doses necessary to ablate gross tumors without causing radiation-induced liver disease (RILD) [11]. However, technical developments of radiation therapy such as three-dimensional conformal radiotherapy (3D-CRT) and intensity modulated radiation therapy have gradually expanded their role from a palliative to a curative intent, whereby high-dose radiation can be delivered to the tumor more safely without affecting the liver function. In recent years, the introduction of stereotactic ablative radiotherapy (SABR) has offered even more intensive tumor dose escalation using a hypofractionated regimen with reduced dose to the normal liver, usually below the threshold dose above which severe RILD is observed. Early result of SABR for liver metastases has promising outcomes and very low complication rates. In this review, we will discuss the recent treatment outcome and toxicity following SABR for liver metastases in CRC. The dose constraint and pattern of failure will also be discussed.

## 2. Definitions

### 2.1. Oligometastases

Until fairly recently, distant metastases have been considered to occur late in the natural history of cancer and represent an incurable state, thereby warranting palliative care only. It is now recognized that some patients of distant metastases with “oligo,” or few sites of metastases, may have isolated sites of metastases. Hellman and Weichselbaum hypothesized the intermediate state of metastases between purely localized and widely metastatic disease and coined the term “oligometastases” in which metastases are limited in number and location [12]. For patients with oligometastases, aggressive local therapies might prolong survival, especially when combined with effective systemic therapy to address occult micrometastases. Oligometastases are clinically distinguished by two scenarios; the* de novo* oligometastases, in which the tumor early in the evolution of metastatic progression producing metastasis that are limited in number and site, and the* induced* oligometastases, which is generated when effective systemic chemotherapy eradicates the majority of metastatic deposits in a patients with wide spread metastatic 2 disease [13]. The induced “oligometastases” group is likely to have a much less favorable prognosis.

### 2.2. Stereotactic Ablative Radiotherapy (SABR)

**Figure 1:**
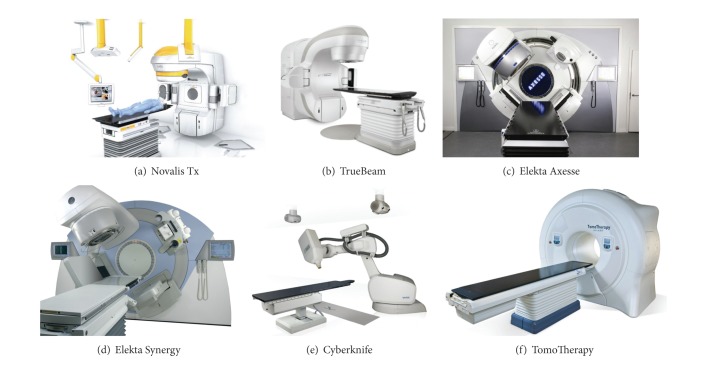
Various treatment devices available for stereotactic ablative radiotherapy.

SABR, also called stereotactic body radiation therapy (SBRT), is an extension of intracranial stereotactic radiosurgery (SRS) that encompasses all sites below the cranium. The American Society of Radiation Oncology defines SABR as external beam radiotherapy used to deliver a high dose of radiation extremely precisely to an extracranial target within the body, as a single dose or a small number of fractions [32]. SABR is used and showed efficacy in controlling early stage primary and oligometastatic disease at a wide range of tumor sites, including lung, liver, pancreas, prostate, spine, and head and neck [33, 34]. In addition to increasing the accuracy of radiation dose delivery, thus reducing irradiated adjacent normal structures, the high radiation dose per fraction can potentially ablate all tissues in the treated area. Because of the high fractional dose, it is extremely important that the treatment organ geometry is as close as possible to the planning CT data. Immobilization devices or other types of interfractional motion management is one way to move towards that goal. The techniques for improving the accuracy and precision can be classified into two broad categories: immobilization and motion reduction techniques and image guidance. Immobilization with stereotactic body frames aims to optimize patient fixation, provide external reference system for stereotactic coordinates, and use a device to reduce breathing mobility. Image guidance technology allows the guidance of dose delivery with 3D real-time information of target localization. These tools serve to reduce patient set-up errors and provide systematic assessment of organ motion and deformation during the course of treatment. Treatment planning is usually based on patients' CT images and includes either multiple noncoplanar fixed gantry beams or arcs. Many different treatment devices are commercially available for SABR including Novalis Tx (Brainlab AG, Feldkirchen, Germany), TrueBeam (Varian Medical Systems, Palo Alto, CA, USA), Elekta Axesse and Synergy (both Elekta, Stockholm, Sweden), Cyberknife (Accuray, Sunnyvale, CA, USA), and TomoTherapy (Accuray, Madison, WI, USA) (Figure 1).

## 3. Selection of Patients for SABR

**Figure 2:**
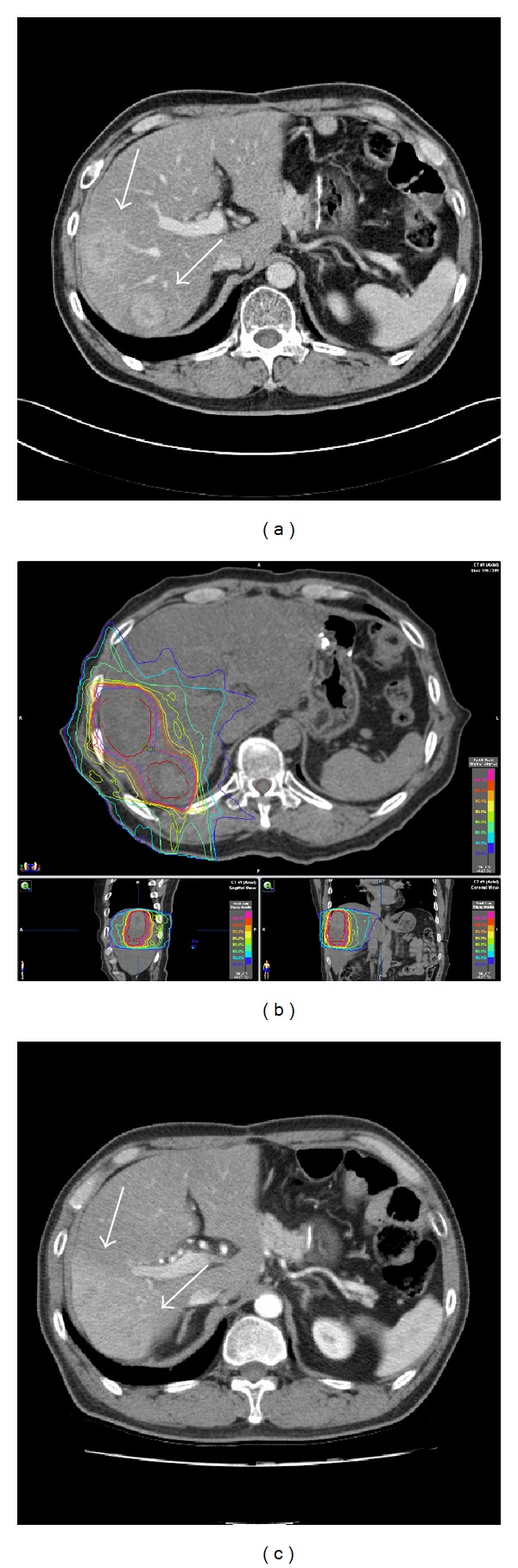
A 52-year-old male patient had been treated with surgery and postoperative adjuvant chemotherapy for sigmoid colon cancer (adenocarcinoma, T2N2M0). 13 months later, liver metastasis developed and he was then treated with salvage chemotherapy; however, follow-up CT scan after the chemotherapy showed progression of liver metastasis (white arrows) (a). We decided to treat him with SABR. The prescriptive dose to the planning target volume including two metastatic tumor lesions was 40 Gy in 4 fractions on consecutive day (b). The CT scan on 3 months after the completion of SABR showed complete response (c). Radiotherapy related change of increased density around the previous tumor lesions was shown but the patient's liver function test was normal.

The main goal of SABR is to achieve local control of each oligometastatic site; however, whether obtaining local control of the metastasis would translate into clinical or survival benefit for the patients depends on multiple factors, including age, performance status, medical comorbidities, and histology of malignancies. Therefore, careful and strict patient selection is needed and the patient's whole condition should be fully considered. Ideal candidates for SABR may be defined as follows: a limited number of metastases (one to five), a limited tumor size (<6 cm), a locally controlled primary site, metachronous occurrence of metastatic disease, favorable histologies (such as CRC and breast cancer), young age, good performance status, at least 700 m of uninvolved liver volume, and adequate pretreatment baseline liver function. In general, the risk of occult diffuse metastases increases as the number of metastases increases, and the best results are found in patients with 3 or fewer metastases, ideally less than 6 cm in maximum diameter [35, 36].  Figure 2 shows the case of good candidate for SABR in liver oligometastases.

## 4. Clinical Outcomes

### 4.1. Retrospective Study

Since the early result was published in 1995 by Blomgren et al. [14], the safety and efficacy of 1–6 fraction SABR have been evaluated in several retrospective series and more recently confirmed in prospective dose escalation results. Several retrospective studies showed good local control rates ranging from 50% to 92%; however, the follow-up times of most studies were relatively short, typically less than 18 months [14–21, 37, 38] (Table 1). The Swedish group, led by Blomgren et al., treated 14 patients with liver metastases (11 CRC, 1 anal canal, 1 kidney, and 1 ovary) with 20–45 Gy in 1 to 4 fractions and reported 95% local control rate with a mean survival rate of 17.8 months on 9.6 months follow-up in the 1998 update. There was no serious toxicity associated with SABR [37]. Wulf et al. from the University of Wuerzburg, Germany, reported 1- and 2-year local control rates of 76% and 61%, respectively, and overall survival rates of 71% and 43%, respectively, after 30 Gy in 3 fractions for 23 liver metastases [38]. No grade 3 or higher toxicities were observed. Update data including 51 patients with liver metastases (majority from CRC, breast, and ovarian cancer) treated with 30–37 Gy in 3 fractions or 26 Gy in a single fraction showed actuarial local control rates of 92% at 1 year and 66% at 2 years [15]. Higher dose was associated with improved local control. There was a trend for patients with CRC liver metastases to have worse local control compared with other histologies. Katz et al. [16] treated 69 patients with 174 liver metastases (CRC 20, breast 16, pancreas 9, and lung 5) with a median dose of 48 Gy in 2–6 fractions and showed 76% and 54% of local control rate on 10 months and 20 months, respectively, with 14.5 months of median survival. There was no grade 3 or higher toxicity.

### 4.2. Prospective Study

There are several prospective trials of SABR for liver oligometastases in the literature and some studies have examined the dose escalation in a formal, structured phase I trial [22–29] (Table 2). Herfarth et al. performed a prospective phase I/II study using single dose SBRT from 14–26 Gy in 33 patients with 56 liver metastases (majority CRC, others from breast cancer, soft tissue sarcoma, lung, pancreas, kidney, or melanoma) [22]. The actuarial local control rates on 6, 12, and 18 months were 75%, 71%, and 67%, respectively. No severe toxicity was observed. In another phase I study of 37.5 Gy in 3 fractions in 17 patients with 34 liver metastases (CRC 14, lung 1, breast 1, and carcinoid 1), Méndez Romero et al. reported 1-year and 2-year local control rates of 100% and 86%, respectively, and 1- and 2-year survival rates of 85% and 62%, respectively, after a median follow-up of 12.9 months [23]. Transient grade 3 elevation of liver enzyme levels was observed within 3 months after treatment in two patients. University of Colorado performed a multi-institutional phase I/II SBRT trial of 47 patients with 63 liver metastases starting with 36 Gy in 3 fractions, and the doses were escalated by 6 Gy per dose up to a defined maximum of 60 Gy. The 2year local control rate was 92% and a median survival rate was 20.5 months [25]. No hepatic toxicity was noted, but grade 3 soft-tissue toxicity was developed in one patient receiving 48 Gy in 3 fractions to the anterior abdominal wall to the region of the subcutaneous tissue. Rule et al. from the University of Texas Southwestern reported on a phase I study using escalated dose from 30 Gy in 3 fractions to 50 Gy in 5 fractions to 60 Gy in 5 fractions. In total, 27 patients with 37 lesions (one to five liver metastases) were enrolled. The median follow-up was 20 months. The 2-year actuarial LC rates were 56%, 89%, and 100% for the cohorts treated with 30 Gy, 50 Gy, and 60 Gy, respectively. There was a significant difference in local control between the cohorts treated with 60 Gy and 30 Gy. No grade 4 or 5 toxic effects or treatment-related grade 3 toxic effects were observed [28]. Lee et al. from the Princess Margaret Hospital conducted phase I/II trial using 6 fractions over weeks of SBRT in 68 patients with liver metastases of varying sizes (up to 3090 mL) to evaluate the safety of SBRT for larger liver metastases [26]. Radiotherapeutic dose was individualized based on the liver volume irradiated in order to avoid RILD (range: 24–60 Gy). With a median follow-up of 11 months, the 1-year LC rate was 71% and the median survival rate was 18 months. There was no RILD, resulting in a low risk of serious liver toxicity (95% CI, 0 to 5.3%). Grade 2 nontraumatic rib fractures occurred in two patients treated with the maximum doses to 51.8 Gy and 66.2 Gy in 6 fractions to 0.5 mL of rib. More recently, Scorsetti et al. published a preliminary result from a phase II trial of high-dose SBRT using 75 Gy in 3 fractions. A total of 61 patients with 76 lesions were treated. With a median follow-up of 12 months, the in-field local response rate was 94%, the median survival rate was 19 months, and 1-year actuarial survival rate was 83.5%. No RILD was detected [29].

Published reports to date have been small and confounded by significant heterogeneity concerning the primary subtype, tumor size and the number of metastases, the number of systemic treatments before and after RT, total dose, and the number of fractions. As a result, it is difficult to evaluate and compare clinical results for liver metastases. Generally, most SABR were performed with 30–60 Gy in 1 to 6 fractions, for 5 or fewer metastases, with maximum tumor sizes of 6 cm, and the reported 2-year local control and survival rates range from 60% to 90% and from 30% to 83%, respectively. Higher dose showed to associate with better local control and a total prescription dose over 48 Gy in 3 fractions was recommended, whenever possible [35]. Other than higher dose, smaller tumor volume, potentially non-CRC metastases, metachronous liver metastases, and the absence of previous systemic chemotherapy were prognostic factors related to improved local control. The possible explanation for better local control in patients with non-CRC metastases than CRC metastases may be that most patients with CRC liver metastases have been heavily treated with systemic and other local treatments before SABR [35].

## 5. Radiation Related Toxicity and Dose Volume Constraints

RILD is the most common hepatic toxicity after liver irradiation, which typically occurs between two weeks to 3 months after RT, presenting with anicteric ascites, hepatosplenomegaly, and elevated alkaline phosphatase [11]. Baseline liver function was found as an important factor in RILD after RT and the tolerance dose of SABR in a cirrhotic liver is less than the tolerance dose of a normally functioning liver [31, 39, 40]. Most patients with metastatic liver tumors have a normally functioning liver, not having underlying cirrhosis or hepatitis, and RILD is rare following SABR [25]. Transient grade 3 elevation of liver enzyme levels occurred in 2 patients treated with 30 to 37.5 Gy in 3 fractions in a phase I/II SABR trial by Méndez Romero et al. [23]. There has been reported 1 hepatic failure 7 weeks after SABR leading to death with median total liver dose of 14.4 Gy and 60% of the liver received >10 Gy in 3 fractions [24]. In a phase I/II study by Lee et al., no RILD was noted in 68 patients who received median mean liver dose of 16.9 Gy in 6 fractions [26]. In a phase I/II study by Rusthoven et al. [25], there was no RILD in 47 patients treated with a critical dose-volume model allowing no more than 700 mL of uninvolved normal liver to receive 15 Gy or greater in 3 fractions in accordance with the Quantitative Analyses of Normal Tissue Effects in the Clinic (QUANTEC) recommendations on liver [31]. Another complication reported after SABR is gastrointestinal and soft-tissue/bone toxicity. Three patients who received a total dose of 30 Gy or higher to the part of the intestine had colonic and duodenal ulcerations; one patient had perforation of a colonic ulceration demanding surgery while two patients had duodenal ulceration [24]. Grade 3 soft-tissue toxicity was observed in 1 patient after receiving 48 Gy in 3 fractions to a region of the subcutaneous tissue [25]. Grade 2 nontraumatic rib fractures occurred in two patients, 6 and 23 months after SABR, who were treated to the maximum dose to 0.5 mL of rib, 51.8 Gy and 66.2 Gy, respectively [26].

Although techniques for delivering large fractions of radiation using SABR to the liver have been widely reported, including various means of body immobilization, stereotactic localization, image guidance, beam arrangements, and dosing techniques, far less is known about the tolerance of the liver or the surrounding critical organs when using large fractions of radiation due to its recent development, fewer patient numbers, and nonuniform reporting of dosimetric parameters. Further clinical data with long follow-up period are needed to ascertain the dose fractionations schedule that optimizes tumor control while minimizing toxicity and to better understand the optimal normal tissue dose volume constraints. When treating the liver, the most relevant normal tissues at risk are the liver, gastrointestinal tract, and chest wall. When gastrointestinal lumen is located in close proximity, it is reasonable to compromise radiation dose or use more protracted fractionation regimens.  Table 3 summarized normal tissue dose constraints for 3-fraction regimen used in clinical trials or recommended by experts.

## 6. Patterns of Failure

A subset of patients with oligometastases has been alive with a prolonged disease-free state; however, most of them eventually developed further metastatic progression following SABR [41]. There are several studies assessing the patterns of failure of oligometastases after local treatment including surgical resection, RFA, cryosurgery, and SABR [41–46]. In a surgical series by Sugihara et al., 60% of patients in whom metastases were confined to the liver from CRC developed recurrence after curative hepatic resection, including 53% in the liver, 31% in the lung, 19% in the abdominal cavity, and 9% in multiple organs [42]. Of those with hepatic recurrence, ten of 34 patients developed tumors at the initial resection bed. Aloia et al. described local and distant hepatic recurrence rate following hepatic resection as 5% and 18%, respectively, in 150 patients with solitary CRC liver metastases [43]. During the median follow-up of 31 months, 58 patients (39%) experienced distant recurrence, 14 in combination with intrahepatic recurrence and 44 with distant-only recurrence. In a clinical investigation on hepatic cryosurgery for CRC oligometastases by Ravikumar et al., the local recurrence rate was 8% in 24 patients during the median follow-up of 2 years [44]. Ten patients (59%) had recurrences in both the remaining liver and extrahepatic sites (lung, peritoneum, and bone), six (35%) in the remaining liver only, and one (6%) in extrahepatic only. In 2 studies comparing the rates and patterns of recurrence following RFA and hepatic resection, the local recurrence rate following RFA (40%–50%) was higher than after resection (<10%) [43, 45]. Although local failure rate was as low as 7.7%, a high rate of new disease was found in a study of RFA by Kosari et al. New intrahepatic disease was seen in 49% of patients, new systemic progression in 24% of patients, and concurrent progression of new intrahepatic and new systemic disease in 22% of patients [46]. Pattern of recurrence after SABR for oligometastases confined to one organ was analyzed by Milano et al. from the University Rochester [41]. 73% of the patients eventually developed new metastases, most frequently occurring in the initially involved organ, but also commonly in other organs. In patients with initial liver oligometastases, the rate of local recurrence was 45%, with 90% of these patients also developing distant recurrence. Common sites of metastatic spread included the liver (95%), other abdominal organs (32%), and lungs (32%).

Although it is difficult to compare the results from these studies, given the heterogeneous nature in patient characteristics and treatment strategies as well as follow-up periods, it seems clear that new metastases to the same organ, the liver, is common regardless of any treatment, as is metastases to other organs, and there is a substantial number of patients who achieve prolonged disease-free survival. Generally around 20% of patients remain disease-free 2–5 years after SABR [34]. This patient group might benefit from further radical intervention to the new metastatic sites, such as resection or further SABR. Some variables such as the initial organ involvement, the use of chemotherapy, the type of local therapy, and primary cancer type, histology, and grade are considered significant in predicting where subsequent metastases are likely to occur. The mechanisms for recurrence in other organs are yet to be fully determined, but genotypic and phenotypic changes that lead to metastatic potential might account for subsequent new metastases.

## 7. Conclusions

SABR is a feasible, safe, and effective treatment for selected patients with oligometastases. Overall, previous studies report local control rates widely ranging from 50% to 100% at one or two years. And actuarial survival at one and two years has been reported ranging from 72% to 94% and from 30% to 62%, respectively, without severe toxicity. The wide variety of treatment techniques and dose fractionation schedules has been reported in the literatures and as yet, there is no consensus on the standard approach to the SABR for liver oligometastases. To find out the best dose fractionation schedules and whether SABR really does improve survival rate, randomized trials will be essential. Although no randomized studies between the major nonsurgical ablative techniques have been completed, a randomized phase III study, comparing RFA and SBAR, is being addressed by a currently ongoing trial in Europe (http://clinicaltrials.gov/show/NCT01233544). The optimal combination of systemic therapy and local therapy is yet to be determined and a few clinical trials integrating systemic therapy with SABR are ongoing. An ongoing phase I/II trial performed by the Princess Margaret Hospital is investigating the use of SABR with sorafenib for the treatment of unresectable liver metastases (http://clinicaltrials.gov/show/NCT00892424). Further investigations are needed to determine whether SABR should be done before, during, or after the systemic therapy session.

## Figures and Tables

**Table 1:** Results of retrospective trials of SABR for liver metastases.

Study	Patients	Lesions	Tumor volume or size (median)	Type of mets	Dose	Median FU	LC	OS	Toxicity
Blomgren et al. (1995) [14]	14	17	3–260 mL	CRC (11) Anal canal (1) Kidney (1) Ovarian (1)	7.7–45 Gy (1–4 fx)	9.6 mo	NR-50% response rate	NR	2 cases of hemorrhagic gastritis

Wulf et al. (2006) [15]	44 (39 liver mets)	51	9–355 mL	CRC (23) Breast (11) Ovarian (4) Other (13)	30–37.5 Gy (3 fx) 26 Gy (1 fx)	2 yr	1-yr LC, 92%, 2-yr LC, 66%	1-yr OS, 72% 2-yr OS, 32%	No grade 2–4 toxicity

Katz et al. (2007) [16]	69	174	0.6–12.5 cm (2.7 cm)	CRC (20) Breast (16) Pancreas (9) Lung (5) Other (19)	30–55 Gy (5–15 fx)	14.5 mo	10-mo LC, 76%, 20-mo LC, 57%	Median survival, 14.5 mo, 6-mo OS, 46%, 12-mo OS, 24%	No grade 3/4 toxicity

van der Pool et al. (2010) [17]	20	31	0.7–6.2 cm (2.3 cm)	All CRC	30–37.5 Gy (3 fx)	26 mo	1-yr LC, 100%, 2-yr LC, 74%	Median survival, 34 mo, 1-yr OS, 100%, 2-yr OS, 83%	2 grade 3 late liver enzyme changes, 1 grade 2 rib fracture

Chang et al. (2011) [18]	65	102	0.6–3088 mL (30.1 mL)	All CRC	22–60 Gy (1–6 fx)	14.4 mo	1-yr LC, 62%, 2-yr LC, 45%	1-yr OS, 72%, 2-yr OS, 38%	2 grade 3 gastritis, 2 grade 3 elevated liver enzymes.

Vautravers-Dewas et al. (2011) [19]	42	62	0.7–10 cm (3.4 cm)		40 Gy (4 fx) 45 Gy (3 fx)	14.3 mo	2-yr LC, 86%	2-yr OS, 48%	

Lanciano et al. (2012) [20]	30 (23 liver mets)	41	2.29–316 mL (60.9 mL)	CRC (15) Breast (3) Esophagus (1) GIST (1) Pancreas (1) NSCLC (2)	36–60 Gy (3 fx) 50 Gy (5 fx)	22 mo	1-yr LC, 92%, 2-yr LC, 56%	1-yr OS, 73%, 2-yr OS, 31%	No grade 3/4 toxicity

Habermehl et al. (2013) [21]	90	138	11–333 mL (62 mL)	CRC (70) Breast (27) Pancreas (11) Ovarian (7) Lung (6) Others (16), by site	10–30 Gy (1 fx)	21.7 mo	87%, 69%, and 59% after 6, 12, and 18 mo	Median OS 24.3 mo; local PFS was 87%, 70%, and 59% after 6, 12, and 18 mo, respectively	No RILD

SABR: stereotactic ablative radiotherapy; LC: local control; OS: overall survival; CRC: colorectal cancer; GIST: gastrointestinal stromal tumor; NSCLC: non-small cell lung cancer; NR: not reported; fx: fractions; RILD: radiation-induced liver disease.

**Table 2:** Results of prospective trials of SABR for liver metastases.

Study	Design	Patients	Lesions	Tumor volume or size (median)	Type of mets	Dose	Median FU	LC	OS	Toxicity
Herfarth et al. (2001) [22]	Phase I/II	33	56	NR by patient	NR by patient (only by lesion)	Dose escalation, 14–26 Gy (1 fx)	18 mo	18-mo LC, 67%	1-yr OS, 72%	No significant toxicity reported

Méndez Romero et al., (2006) [23]	Phase I/II (HCC and mets)	25 (17 liver mets)	34	1.1–322 mL (22.2 mL)	CRC (14) Lung (1) Breast (1) Carcinoid (1)	30–37.5 Gy (3 fx)	12.9 mo	1-yr LC, 100% 2-yr LC, and 86%	1-yr OS, 85%, 2-yr OS, 62%	2 transient grade 3 elevated liver enzymes.

Hoyer et al. (2006) [24]	Phase II (CRC oligomets)	64 (44 liver mets)	NR	1–8.8 cm (3.5 cm)	CRC (44)	45 Gy (3 fx)	4.3 yr	2-yr LC, 79% (by tumor) and 64% (by patient)	2-yr OS, 38%	1 liver failure, 2 severe late GI toxicities

Rusthoven et al. (2009) [25]	Phase I/II	47	63	0.75–97.98 mL (14.93 mL)	CRC (15) Lung (10) Breast (4) Ovarian (3) Esophageal (3) HCC (2) Other (10)	Dose escalation, 36–60 Gy (3 fx)	16 mo	1-yr LC, 95% 2-yr LC, 92%	Median survival, 20.5 mo, 2-yr OS, 30%	No RILD 1 grade 3 soft tissue toxicity

Lee et al. (2009) [26]	Phase I/II	68	140	1.2–3,090 mL (75.9 mL)	CRC (40) Breast (12) Gallbladder (4) Lung (2) Anal canal (2) Melanoma (2) Other (6)	Individualized dose, 27.7–60 Gy (6 fx)	10.8 mo	1-yr LC, 71%	Median survival, 18 mo	No RILD 10% grade 3/4 acute toxicity, no grade 3/4 late toxicity

Ambrosino et al. (2009) [27]	Prospective cohort	27	1–3 lesions for each patient	20–165 mL (69 mL)	CRC (11) Other (16)	25–60 Gy (3 fx)	13 mo	Crude LC rate 74%		No serious toxicity

Rule et al. (2011) [28]	Phase I	27	37	NR		Dose escalation, 30 Gy (3 fx), 50 Gy (3 fx), 60 Gy (5 fx)	20 mo	24-mo LC: (30 Gy) 56%; (50 Gy ) 89%; (60 Gy ) 100		No serious toxicity

Scorsetti et al. (2013) [29]	Phase II	61	76	1.8–134.3 mL (18.6 mL)	CRC (29) Breast (11) GY (7) Other (14)	75 Gy (3 fx)	12 mo	12-mo LC 94%	Median survival, 19 mo; 12-mo OS, 83.5%; 18-mo OS, 65%	No RILD

SABR: stereotactic ablative radiotherapy; LC: local control; OS: overall survival; CRC: colorectal cancer; HCC: hepatocellular carcinoma; GY: gynecological; NR: not reported; fx: fractions; RILD: radiation-induced liver disease.

**Table 3:** Summary of dose volume constraints for 3-fraction SABR regimen.

Organs at risk	Wulf et al. [15]	Hoyer et al. [24]	Timmerman [30]	Rusthoven et al. [25]	QUANTEC [31]
Liver	*D*_30%_ < 21 Gy *D*_50%_ < 15 Gy	700 mL < 15 Gy	700 mL < 17 Gy	700 mL < 15 Gy	700 mL ≤ 15 Gy *D*_mean_ < 15 Gy

Esophagus	*D*_5 mL_ < 21 Gy	*D*_1 mL_ < 21 Gy	*D*_5 mL_ < 21 Gy, *D*_max⁡_ < 27 Gy	NA	NA

Stomach	*D*_5 mL_ < 21 Gy	*D*_1 mL_ < 21 Gy	*D*_10 mL_ < 21 Gy *D*_max⁡_ < 24 Gy	*D*_max⁡_ ≤ 30 Gy	*D*_max⁡_ < 30 Gy

Bowel	*D*_5 mL_ < 21 Gy	*D*_1 mL_ < 21 Gy	*D*_5 mL_ < 16 Gy *D*_max⁡_ < 24 Gy	*D*_max⁡_ ≤ 30 Gy	*D*_max⁡_ < 30 Gy

Kidney	NA	Total kidney *D*_35%_ < 15 Gy	Total kidney 200 mL < 14.4 Gy	Total kidney *D*_35%_ < 15 Gy	NA

Spinal cord	NA	*D*_max⁡_ < 18 Gy	*D*_0.25 mL_ < 18 Gy *D*_max⁡_ < 22 Gy	*D*_max⁡_ ≤ 18 Gy	*D*_max⁡_ ≤ 20 Gy

Heart	*D*_5 mL_ < 21 Gy	*D*_1 mL_ < 30 Gy	*D*_15 mL_ < 24 Gy *D*_max⁡_ < 30 Gy	NA	NA

SABR: stereotactic ablative radiotherapy; QUANTEC: Quantitative Analyses of Normal Tissue Effects in the Clinic; NA: not available; *D*_*x*%_: dose to *x*%; *D*_*x* mL_: dose to *x* mL; *D*_max⁡_: maximum point dose; *D*_mean_: mean dose.
